# Additive Effect of Metabolic Syndrome on Brain Atrophy in People Living with HIV–Magnetic Resonance Volumetry Study

**DOI:** 10.3390/metabo14060331

**Published:** 2024-06-13

**Authors:** Vanja Andric, Jasmina Boban, Daniela Maric, Dusko Kozic, Snezana Brkic, Aleksandra Bulovic

**Affiliations:** 1 Department for Infectious Diseases, Faculty of Medicine Novi Sad, University of Novi Sad, Hajduk Veljkova 3, 21000 Novi Sad, Serbia; jasmina.boban@mf.uns.ac.rs (J.B.); daniela.maric@mf.uns.ac.rs (D.M.); dusko.kozic@mf.uns.ac.rs (D.K.); snezana.brkic@mf.uns.ac.rs (S.B.); aleksandra.bulovic@mf.uns.ac.rs (A.B.); 2 Clinic for Infectious Diseases, Clinical Center of Vojvodina, Hajduk Veljkova 1, 21000 Novi Sad, Serbia; 3 Department for Radiology, Oncology Institute of Vojvodina, Put dr Goldmana 4, 21204 Sremska Kamenica, Serbia

**Keywords:** HIV, metabolic syndrome, brain atrophy, volumetry

## Abstract

With people living with HIV (PLWH) reaching the senium, the importance of aging-related comorbidities such as metabolic syndrome (MS) becomes increasingly important. This study aimed to determine the additive effect of MS on brain atrophy in PLWH. This prospective study included 43 PLWH, average age of 43.02 ± 10.93 years, and 24 healthy controls, average age of 36.87 ± 8.89 years. PLWH were divided into two subgroups: without MS and with MS, according to NCEP ATP III criteria. All patients underwent brain magnetic resonance imaging (MRI) on a 3T clinical scanner with MR volumetry, used for defining volumes of cerebrospinal fluid (CSF) spaces and white and grey matter structures, including basal ganglia. A Student’s *t*-test was used to determine differences in brain volumes between subject subgroups. The binary classification was performed to determine the sensitivity and specificity of volumetry findings and cut-off values. Statistical significance was set at *p* < 0.05. PLWH presented with significantly lower volumes of gray matter, putamen, thalamus, globus pallidus, and nc. accumbens compared to healthy controls; cut-off values were: for gray matter 738.130 cm^3^, putamen 8.535 cm^3^, thalamus 11.895 cm^3^, globus pallidus 2.252 cm^3^, and nc. accumbens 0.715 cm^3^. The volumes of CSF and left lateral ventricles were found to be higher in PLWH with MS compared to those without MS, where, with a specificity of 0.310 and sensitivity of 0.714, it can be assumed that PLWH with a CSF volume exceeding 212.83 cm^3^ are likely to also have MS. This suggests that PLWH with metabolic syndrome may exhibit increased CSF volume above 212.83 cm^3^ as a consequence of brain atrophy. There seems to be an important connection between MS and brain volume reduction in PLWH with MS, which may add to the accurate identification of persons at risk of developing HIV-associated cognitive impairment.

## 1. Introduction

In the Republic of Serbia, from the onset of the HIV epidemic in 1985 up to 2022, according to data from the Institute of Public Health of Serbia’s “Dr Milan Jovanović Batut”, a total of 4524 individuals were registered as HIV-infected. By the end of 2021, official records indicated 3045 individuals living with diagnosed HIV infection in Serbia, with an estimated 550 individuals still unaware of their HIV-positive status [1]. The main result of the introduction of cART (combined antiretroviral therapy) is effective peripheral suppression of viral replication and the consequent prolongation of life expectancy in patients with HIV infection. However, the latency of HIV in its reservoirs—adipocytes, microglia, and macrophages—induces a persistent systemic low-level inflammation and immune activation, leading to a series of comorbidities [2,3,4]. Adipocyte latency is reflected through an intensive process of lipolysis, hyperglycemia, and hypertriglyceridemia, which results in the redistribution of adipose tissue in the body and the accumulation of visceral fat [5,6]. Visceral fat itself is the source of many inflammatory proteins and, together with HIV infection, it contributes to a number of complications at the cardiometabolic, neurocognitive, and many other levels [7].

Several studies have shown that patients with HIV infection presented lower brain volumes compared to healthy controls [8]. The degree of brain tissue atrophy in HIV infection is variable, with recent studies showing the presence of brain atrophy even in neuroasymptomatic patients [9]. In addition, people with metabolic syndrome (MS), steatosis, elevated cardiovascular risk, etc. also have significantly lower brain volumes than healthy controls [10,11,12,13,14]. The additive effect of HIV infection and MS as comorbidity on the reductive changes in the central nervous system has not been evaluated to date.

This study aimed to determine whether there was a significant additive effect of metabolic syndrome as comorbidity on brain atrophy in patients with HIV infection.

## 2. Materials and Methods

### 2.1. Subject Selection

This cross-sectional study included a total of 24 healthy subjects, average age of 36.87 ± 8.89, and 43 HIV-positive male subjects, an average age 43.02 ± 10.93 years. HIV-positive subjects were further divided into subgroups of HIV-positive patients without the presence of metabolic syndrome, and HIV-positive patients with diagnosed metabolic syndrome, according to NCEP ATP III criteria [15]. All HIV-positive patients were stable on cART for at least 6 months, with undetectable viral loads. At the time of the study, all patients included were on the “old fashioned cART” (combination of two drugs from the non-nucleoside reverse transcriptase inhibitor group, and one drug from the protease inhibitors group), that did not include integrase strand transfer inhibitors (INSTI). All subjects underwent screening of neurocognitive testing using the Mini-Mental State Examination (MMSE) and International HIV-dementia score for the exclusion of manifest cognitive disorder.

### 2.2. Inclusion Criteria for the Study

- In the group of healthy subjects, inclusion criteria were:
  1. Age over 18
  2. Absence of any pathological findings in clinical examination and laboratory results
- In the groups of HIV-positive patients, inclusion criteria included: the presence of HIV infection confirmed by Western blot analysis, and:
  1. Absence of any pathological findings in clinical examination and laboratory results for the subgroup of patients with HIV infection without comorbidities.
  2. Confirmed metabolic syndrome using NCEP ATP III criteria, and the absence of any other pathological findings on clinical examination and laboratory results for the group of patients with HIV infection with metabolic syndrome.

### 2.3. Exclusion Criteria from the Study

- In the group of healthy subjects, the exclusion criteria were:
  1. The presence of focal or diffuse white mass lesions (brain tumors, metastases, vascular malformations)
  2. History of brain irradiation
  3. Active substance abuse and alcohol abuse
  4. Manifest neurocognitive disorder confirmed by Mini-Mental State Examination (MMSE) and International HIV-dementia score tests
- In the groups of HIV patients, the exclusion criteria were:
  1. The presence of active opportunistic CNS infection (toxoplasmosis, cytomegalovirus, tuberculosis, PML [progressive multifocal leukoencephalopathy], cryptococcosis)
  2. The presence of focal or diffuse white mass lesions (brain tumors, metastases, vascular malformations)
  3. History of brain irradiation
  4. Active substance abuse and alcohol abuse
  5. Co-infections with Hepatitis C and Hepatitis B
  6. Manifest neurocognitive disorder confirmed by Mini-Mental State Examination (MMSE) and International HIV-dementia score tests
  7. Contraindications for MR imaging. The study received approval from the institutional ethics committees, and all participants provided informed consent prior to their inclusion.
    - Clinical and laboratory data
    - Blood glucose, high-density lipoprotein (HDL), low-density lipoprotein (LDL), and triglycerides (TG) levels were obtained from each patient. Body height (BH), waist (WC) and hip circumference (HC), waist-to-hip ratio (WHR), BMI (body mass index), and blood pressure were also measured for each patient.
    - MR imaging

All patients underwent conventional MRI on a 3T magnetic resonance unit (Trio Tim, Siemens, Erlangen, Germany) using an 8-channel head coil. Conventional MRI imaging consisted of T1-weighted sagittal spin echo (TR/TE 440 ms/3.8 ms, section thickness 5 mm, duration 2:00 min), T2-weighted turbo spin echo axial (TR/TE 5150 ms/105 ms, section thickness 5 mm, duration 2:57 min), FLAIR (Fluid Attenuation Inversion Recovery) axial tomograms (TR/TE 8000 ms/101 ms, 5 mm section thickness, duration 3:30 min), diffusion-weighted imaging (DWI) (TR/TE4100 ms/91 ms, 5 mm section thickness, duration 2:07 min), coronal T2-weighted turbo spin echo tomograms (TR/TE 7150 ms/111 ms, section thickness 5 mm, duration 2:17 min), and 3D T1W MP-RAGE (magnetization prepared rapid acquisition gradient echo) sagittal tomograms (TR/TE 1530 ms/2.97 ms, section thickness 1 mm, duration 5:12 min).

Volumetric measures of the brain structures for all subjects were obtained using an online automatic analysis system called “VolBrain”, available through the website: www.volbrain.upv.es (accessed on 23 September 2019). Raw data were converted to NIFTI (the neuroimaging informatics technology initiative) format from DICOM (digital imaging and communications in medicine) using a dcm2nii program (part of the MRICRON software version 1.0.20190902), available at: http://people.cas.sc.edu/rorden/mricron/index.html (accessed on 23 September 2019). The VolBrain system displays volumes and percentages of structural asymmetry at the levels of the intracranial cavity (ICC defined as the sum of all white and gray mass and cerebrospinal fluid [CSF]), tissue volumes: white mass, gray mass, and CSF volume, volumes of cerebrum, cerebellum, and brain stem (with separation of right and left cerebral and cerebellar hemispheres), lateral ventricles, and subcortical structures of gray matter (putamen, nucleus caudatus, globus pallidus, thalamus, hippocampus, amygdala, and nucleus accumbens).

### 2.4. Statistical Analysis

Statistical data processing was done in the software program SPSS v23.0. The normality of the distribution and the homogeneity of the variance was checked for each group, and afterwards, appropriate statistical analyses were performed. To determine the effects of metabolic syndrome on volumetric findings in patients with HIV infection and metabolic syndrome compared to patients with HIV infection only, an ANOVA variance analysis was performed, with post hoc significance testing between subgroups. For every statistically significant result, binary classification analysis was performed to determine the sensitivity and specificity of volumetry, and cut off values were defined. The values of *p* < 0.05 were considered statistically significant.

## 3. Results

### 3.1. Demographic and Clinical Data of the Subjects

Demographic and clinical data of the subjects are summarized in Table 1. The average age of healthy subjects was 46.87 ± 8.89. The average age of HIV-positive patients was 43.02 ± 10.93 years, the average duration of infection was 6.40 ± 4.75, and the average duration of therapy was 5.98 ± 4.28. There was no statistically significant difference in the average age between healthy subjects and HIV-positive patients (t = 1.473, df(64), *p* > 0.05). Average values of immunological parameters of HIV-positive patients are also summarized in Table 1. Furthermore, patients with chronic HIV infection were divided into two groups: patients with and without metabolic syndrome. Their clinical and demographic characteristics are summarized in Table 2. In the group of HIV-positive patients with MS, the average age was 48.36 ± 8.44, average duration of infection was 10.64 ± 6.67, and the average duration of therapy was 9.00 ± 4.50. In the group of HIV-positive patients without metabolic syndrome, the average age was 43.68 ± 9.00, the average duration of infection was 9.55 ± 4.49, and the average duration of therapy was 8.41 ± 3.51. An independent *t*-test showed that there were no statistically significant differences between any of the characteristics in the two subgroups of HIV-positive patients (Table 2). There were no significant differences in the duration of HIV infection and cART therapy in the two subgroups of HIV-positive patients.

### 3.2. MR Volumetry Differences between Healthy Subjects and HIV-Positive Patients

The first part of the analysis was directed towards the examination of brain MR volumetry differences between healthy subjects and HIV-positive patients. With the goal of determining differences in volumetry findings between healthy controls and patients with HIV infection, a series of t-tests for independent samples were conducted. HIV-positive patients presented with significantly lower volumes of gray matter, putamen, thalamus, globus pallidus, and nc. accumbens (Table 3).

ROC curve analysis showed that the value for area under the curve, AUC ranged from 0.654 to 0.750, was statistically significant for each brain region. The obtained values were considered as good (above 0.7 in the case of putamen and globus pallidus) or satisfying (above 0.6 in the case of gray matter, thalamus, and nc. accumbens), and it can be concluded that having HIV can predict MR volumetry findings (Table 4).

**Figure 1:**
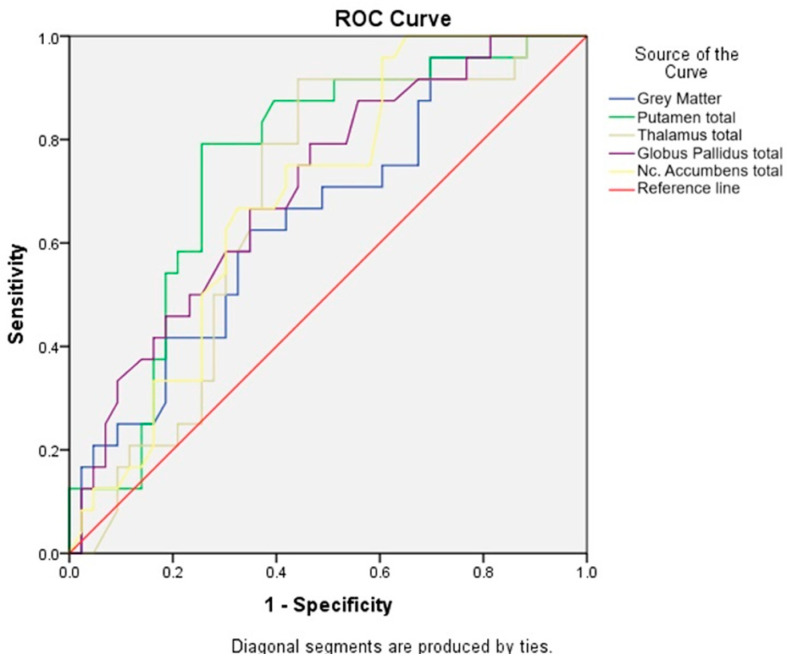
Sensitivity of the test for healthy subjects and HIV-positive patients.

Cut-off values were determined using Youden’s J index [16], which is the most common method that combines sensitivity and specificity into a single measure. It is calculated as Sensitivity + Specificity − 1, and it is equivalent to the maximum vertical distance from no discrimination line to the curve. Based on ROC curve values of volumetry defined off score, the values in gray matter are 738.130 cm^3^, in putamen (total) 8.535 cm^3^, in thalamus (total) 11.895 cm^3^, in globus pallidus (total) 2.252 cm^3^, and for nc. accumbens (total) 0.715 cm^3^ (Figure 1).

### 3.3. MR Volumetry Brain Differences between HIV-Positive Patients with and without Metabolic Syndrome

The second part of the analysis was directed toward the examination of brain MR volumetry differences between HIV-positive patients with and without metabolic syndrome. For this purpose, an ANOVA was conducted. Results showed that the volumes of cerebrospinal fluid (CSF) and lateral ventricles were higher in patients with metabolic syndrome (Table 5). Variance analysis was conducted to determine differences in volumetry findings between the left and right ventricles. The left ventricle was of significantly higher volume (Table 6).

**Figure 2:**
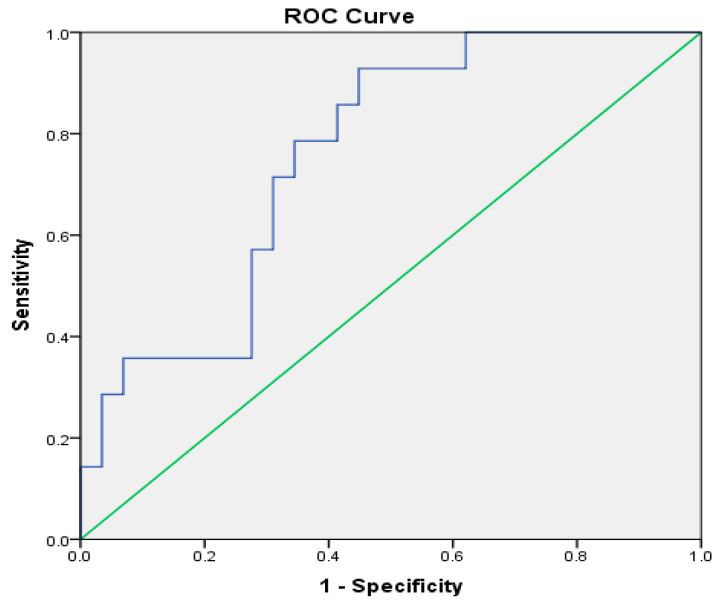
Sensitivity of the volumetry values of CSF in patients with HIV infection and metabolic syndrome.

In determining the specificity and sensitivity of volumetry findings of the left ventricle, the cut-off value could not be identified, considering that the ROC curve value is 0.671 and the model was not statistically significant (Table 7). On the other hand, based on the statistically significant model with high specificity and sensitivity, it can be assumed that CSF volume can successfully predict the presence of metabolic syndrome in PLWH. Based on Youden’s J Index, used for determining cut-off scores, patients with a CSF volume above 212.83 cm^3^ will have metabolic syndrome (sensitivity-0.714, specificity-0.310) (Table 8, Figure 2).

## 4. Discussion

The introduction of combined antiretroviral therapy and effective suppression of the virus in the peripheral compartment led to a decrease in the incidence of AIDS-related diseases. However, the incidence of HIV-associated neurocognitive disorder remains stable [15]. A number of studies so far have illustrated the reduction of brain volume tissues in PLWH [17,18]. Although the processes of neurodegeneration in PLWH are diffuse, the most affected areas in which brain tissue reduction have been registered are the gray matter, as well as the basal ganglia [19,20]. In our study, PLWH compared to healthy subjects had decreased volumes of gray matter, putamen, thalamus, globus pallidus, and nc. Accumbens, which are the results consistent with many previous studies [8,17,19,21]. Bearing in mind that HIV-associated neurocognitive disorder (HAND) is considered to be subcortical dementia characterized by disorders of attention and memory, verbal and motor abilities, as well as sensory modalities, perception, and abstract thinking [15], the verified reduction of brain tissue in the above-mentioned regions in our research is expected. The underlying mechanism of brain injury in PLWH refers to the mechanism of continuous low-level inflammation and immune activation that takes place despite the effective suppression of the virus. The etiological background is dual, and it implies continuous inflammation that leads to increased permeability of the blood–brain barrier, and on the other hand, persistent inflammation at the level of brain tissue that potentiates the damage [3,13,22]. A growing number of recent studies focused on neurodegenerative processes in chronic HIV infection are directed towards the assumption that PLWH have accentuated aging, which implies a third contributing factor to the development and progress of neurocognitive disorders associated with HIV infection [23,24,25]. As already mentioned, the early introduction of cART (practically immediately after seropositivity confirmation) resulted in the prolonged lifespan of PLWH and more prevalent non-HIV-related disorders in this population. Among those, the most important are those associated with systemic inflammation—metabolic syndrome, hepatic steatosis/non-alcoholic liver disease, and cardiovascular disorders [4,5,6,7,26]. The incidence of metabolic syndrome (MS) in patients living with HIV (PLWH) compared to the general population is not clearly defined, probably due to the overlapping of host, viral, and antiretroviral therapy factors that solely or in synergy contribute to the components of this syndrome. Recent studies reported the prevalence of MS in PLWH from 10% to over 50%, depending on the studied population and region [24,27,28,29]. However, the relationship between MS and brain atrophy is a recently recognized issue that is not clearly understood nor explained. Our results showed a significant increase in the volume of CSF and lateral ventricle in HIV-positive patients with MS compared to HIV-positive subjects without defined metabolic syndrome. There was no significant difference in the mean age of patients in observed cohorts, so the effect of age on brain reductive changes can be neglected. Additionally, no significant differences in the duration of HIV infection or the time from the cART initiation were present between the two subgroups of HIV-positive patients, which is an important fact given that chronically HIV-infected individuals on stable cART undergo progressive loss of brain tissue and present with changes on MR volumetry of the brain [20]. Despite the undetectable plasma viral load in HIV-positive patients on cART, HIV remains latent in several cell types, among which the adipocytes play an important role. Adipocytes continuously secrete adipokines—inflammation mediators—that have systemic activity both in peripheral and central nervous system compartments [30]. Activated adipocytes via chemokines and cytokines induce monocyte activation that reaches the brain. Recent studies showed continuous microglia receptor expression in chronic HIV infection. This activation is induced by inflammatory cytokines and chemokines released from adipose tissue that further induce the secretion of correspondent mediators in the central nervous system. Furthermore, this induces the astrocyte activation and neuronal apoptosis [30,31,32]. As previously mentioned, HIV-induced neurodegeneration and astrocyte activation in metabolic syndrome can have synergistic effects, but to which point these processes are additive remains to be explained. The results of our study showed that the volume of CSF spaces in HIV-positive patients with MS was significantly higher than in HIV-positive patients without MS. This might speak in favor of the additive effect of HIV infection and MS on the brain of HIV-positive patients. Moreover, our results showed that in patients with CSF volumes over 2012.82 cm^3^, the presence of concomitant metabolic syndrome can be assumed with high specificity. The potential underlying mechanism of the influence of HIV infection and metabolic syndrome on CSF would include persistent low-level inflammation in the brain at the level of the hematoencephalic barrier, multiplied by the effect of the metabolic syndrome on the small vessels in the white matter [33]. This finding should induce additional evaluation of the patient in order to reduce potential complications of both disorders. Timely evaluation of these subgroups of patients is important from the aspect of treatment and prevention of complications that significantly degrade the quality of life of these patients. One interesting result of our study was a significant reduction of the volume of the left ventricle. In their recent study, Li et al. showed that a lower CD4+/CD8+ ratio was associated with higher volumes of the left ventricle and the whole ventricular system [34]. However, in our study, no obvious correlations were found with the serological parameters of the HIV infection. In the context of the previous findings, it is important to note that the study by Li et al. demonstrated differences in ventricular volumes solely between individuals with asymptomatic neurocognitive impairment (ANI) and normal controls. Subjects who did not meet the criteria for ANI and cognitively intact HIV-positive individuals did not exhibit significant differences in brain volumes compared to control subjects. Specifically, among these patients, a lower CD4+/CD8+ ratio was correlated with larger left ventricular and ventricular system volumes, which could potentially be attributed to persistent inflammation and immunosenescence induced by viral infection. However, the impact of these serological parameters was not observed when analyzing all subjects collectively [34]. Most of the studies confirming the relationship between ventricular volume and serological parameters, such as current CD4+ levels [35] have been conducted on large population cohorts, each comprising over 1000 subjects. However, our study population is comparatively small, and there is a risk that the potential significance of our findings might be obscured by the limited sample size. Furthermore, in a paper from the CHARTER study, higher CSF ventricular volumes were associated with the presence of diabetes, which is in line with our results [36]. However, over a third of subjects involved in the CHARTER study were cognitively impaired; to the contrary, our study group did not include patients with international HIV Dementia Scale scores lower than 10 [37]. This study has several limitations. Firstly, the study sample size is relatively small, and the study was conducted in a cross-sectional manner. However, strict exclusion criteria were applied, and subject selection was meticulously executed, leading to the exclusion of a certain number of patients. Conducting longitudinal studies would enable the tracking of patients over time, thereby aiding in the understanding of the pathophysiological mechanisms underlying the synergy between HIV and metabolic syndrome in the development of neurocognitive impairment and brain atrophy.

Moreover, implementing longitudinal study designs and including a larger number of participants would allow for the incorporation and monitoring of other parameters influencing metabolic syndrome, such as lifestyle factors.

Lastly, the type of combined antiretroviral therapy (cART) used is crucial for interpreting the results. In our study sample, at the time of the study, a considerable proportion of patients were on a cART regimen that did not include integrase strand transfer inhibitors (INSTIs). A follow-up study could be a reasonable approach to explore the findings in these patients who have now transitioned to modern cART regimens.

## 5. Conclusions

Neurodegenerative processes in HIV infection are diffuse and constant, despite the early introduction of cART and effective peripheral suppression of viral replication. Due to that, the incidence of HAND remains stable and, along with comorbidities that are now the leading cause of death in PLWH, represent an emerging problem in the treatment of people living with HIV. Brain volumetric analysis can reliably point to the presence of metabolic syndrome in HIV-positive patients and vice versa. This result is expected since many factors associated with the infection itself and the application of cART contribute to the development of metabolic syndrome, in addition to the classical risk factors. There seems to be an important connection between metabolic syndrome and the presence of volume reduction in the brain. The global volume reduction in the brain is associated with the enlarged CSF spaces (and lateral ventricles) and is more prominent in HIV-positive patients with metabolic syndrome, according to the results of our study. This identifies these persons as those at risk of developing HIV-associated cognitive impairment and may point to the elevated risk of impairment in executive, cognitive, and emotional functioning. That way, after diagnosing metabolic syndrome in an HIV-positive patient, the clinician should prompt a detailed neurocognitive evaluation of the patient in order to exclude signs of cognitive disorder in time.

## Figures and Tables

**Table 1:** Demographic and clinical characteristics of participants.

Category	N	X ± SD
Average age of HIV-positive patients (years)	43	43.02 ± 10.93
Average age of healthy subjects (years)	24	46.87 ± 8.89
Duration of infection (years)	43	6.40 ± 4.75
Duration of therapy (years)	43	5.98 ± 4.28
nadirCD4+ (cells/mm^3^)	43	293.82 ± 189.79
CD4+ (cells/mm^3^)	43	580.20 ± 227.65
CD8+ (cells/mm^3^)	43	943.69 ± 502.93
CD4+/CD8+index	43	0.47 ± 0.43
IL-6 (pg/mL)	43	2.91 ± 2.44
TNFα (pg/mL)	43	3.28 ± 1.02

**Table 2:** Demographic and clinical characteristics of PLWH.

Category	N	X ± SD	*p*
Average age of PLWH without MS (years)	29	43.68 ± 9.00	>0.05
Average age of PLWH with MS (years)	14	48.36 ± 8.44	
Average duration of infection of PLWH without MS (years)	29	9.55 ± 4.49	>0.05
Average duration of infection of PLWH with MS (years)	14	10.64 ± 6.67	
Average duration of therapy of PLWH without MS (years)	29	8.41 ± 3.51	>0.05
Average duration of therapy of PLWH with MS (years)	14	9.00 ± 4.50	

**Table 3:** Volumetry findings between healthy subjects and PLWH.

Volume (cm^3^)	HIV	N	M	SD	t	df	*p*
Gray matter	positive	43	706.19	99.77	−2.091	65	<0.05
	negative	24	752.89	59.44			
Putamen–total	positive	43	8.17	1.99	−2.855	65	<0.01
	negative	24	9.40	0.87			
Thalamus–total	positive	43	11.06	3.14	−2.059	65	<0.05
	negative	24	12.42	0.98			
Globus pallidus–total	positive	43	2.29	0.59	−2.502	65	<0.05
	negative	24	2.62	0.29			
Nc. Accumbens–total	positive	43	0.63	0.20	−2.579	65	<0.05
	negative	24	0.74	0.10			

**Table 4:** Sensitivity and specificity of the test for healthy subjects and HIV-positive patients.

Region	AUC	Std. Error	Asymptotic Sig.	Asymptotic 95% Confidence Interval	
				Lower Bound	Upper Bound
Gray matter	0.654	0.069	0.038	0.519	0.788
Putamen total	0.750	0.062	0.001	0.630	0.871
Thalamus total	0.677	0.067	0.017	0.546	0.808
Globus pallidus total	0.706	0.065	0.005	0.580	0.833
Nc. Accumbens total	0.688	0.064	0.011	0.562	0.813

**Table 5:** Differences in volumetry findings relative to the presence of metabolic syndrome.

Volume (cm^3^)	Met. Syndrome	M	SD	F	*p*
CSF	no	191.164	63.251	8.823	0.005
	yes	252.643	64.341		
Lateral ventricle total	no	12.565	9.580	4.343	<0.05
	yes	19.736	12.447		

**Table 6:** Differences in volumetry findings between left and right ventricle in patients with HIV infection and metabolic syndrome.

Volume (cm^3^)	Met. Syndrome	M	SD	F	*p*
Right lateral ventricle	no	6.219	5.600	3.456	>0.05
	yes	9.645	5.792		
Left lateral ventricle	no	6.345	4.347	4.827	<0.05
	yes	10.091	6.773		

**Table 7:** Specificity and sensitivity of the volumetry values of the left ventricle in patients with HIV infection and metabolic syndrome.

Structure	Area	Std. Error	Asymptotic Sig.	Asymptotic 95% Confidence Interval	
				Lower Bound	Upper Bound
Left lateral ventricle	0.671	0.085	0.072	0.504	0.838

**Table 8:** Specificity and sensitivity of the volumetry values of CSF in patients with HIV infection and metabolic syndrome.

Structure	Area	Std. Error	Asymptotic Sig.	Asymptotic 95% Confidence Interval	
				Lower Bound	Upper Bound
CSF	0.756	0.074	0.007	0.612	0.901

## Data Availability

The data that support the findings of this study are available on request from the corresponding author, VA. The data are not publicly available due to containing information that could compromise the privacy of research participants.
